# Relationship Between Poor Sleep Quality and Body Mass Index Among University Students at Imam Mohammad Ibn Saud Islamic University

**DOI:** 10.7759/cureus.80327

**Published:** 2025-03-10

**Authors:** Rakan H Khushaim, Abdalaziz B Alyousef, Meshaal A Alqhtani, Ahmed S Almutawa, Khalid A Bin Abdulrahman

**Affiliations:** 1 College of Medicine, Imam Mohammad Ibn Saud Islamic University, Riyadh, SAU; 2 Family and Community Medicine, College of Medicine, Imam Mohammad Ibn Saud Islamic University, Riyadh, SAU

**Keywords:** body mass index (bmi), cognitive effects, cross-sectional, obesity, psqi, saudi arabia, sleep disturbance, sleep quality, university students, weight fluctuations

## Abstract

Introduction

A chronic lack of sleep can lead to physical exhaustion, negatively affecting university students' academic performance. The relationship between insufficient sleep and increased body mass index (BMI) has garnered significant attention. Obesity, weight fluctuations, and cognitive conditions contribute to poor sleep quality.

Aim

The aim of this study was to investigate the correlation between poor sleep quality and BMI, which may negatively impact academic performance among university students at Imam Mohammad Ibn Saud Islamic University (IMSIU) in Riyadh, Saudi Arabia. The study also explores whether changes in weight and cognitive status are linked to poor sleep quality.

Subject and methods

This cross-sectional study was conducted among 1297 university students from IMSIU. A self-administered questionnaire was distributed among students using an online survey. The questionnaire included sociodemographic questions, sleep quality assessed through the Pittsburgh Sleep Quality Index (PSQI), and four subjective questions regarding weight and cognitive status changes over the last month. The study spanned from September 2021 to May 2023. The study utilized the chi-square test and a subsequent multivariate analysis to examine the correlation between poor sleep quality and high BMI, weight changes, and cognitive effects among students.

Results

The 1297 students were from various disciplines under science and humanities; 70.8% were females, and 48% were aged between 17 and 20 years old. Around 13.6% of students were obese, and 85.3% had sleep problems. In univariate analysis, the factors that influence poor sleep quality were high BMI, experiencing weight loss or weight gain, and experiencing cognitive effects like difficulty concentrating. In a multivariate regression model, experienced weight loss, weight gain, and cognitive effects were identified as the significant independent predictors of poor sleep.

Conclusion

The incidence of poor sleep among university students was high. The study identified increased BMI, weight loss, weight gain, and cognitive dysfunction due to lack of sleep as the influential factors in poor sleep quality. This study highlights the need for targeted interventions to improve sleep hygiene and promote physical well-being programs in this population.

## Introduction

Lack of sleep is defined as having insufficient duration and/or quality of sleep to maintain reasonable alertness, performance, and health. Adequate, high-quality sleep for an appropriate period aids memory processing and learning. It improves concentration, executive cognitive functioning, sensorimotor integration, and memory processing [1,2].

Evidence has grown over the past decade, alluding to the role of short sleep hours as a novel factor of risk for an increased body mass index (BMI) and feelings of fatigue, which may contribute to lower physical activities. The rise in weight has grown to epidemic proportions. Similarly, there has been a parallel epidemic of chronic sleep deprivation. According to annual surveys done by the National Sleep Foundation, 35% of American adults by the year 1998 were obtaining eight hours of sleep, and the percentage went down to 26% by 2005 [2-4].

Sleep quality is crucial for an individual's health and optimal body function. A consistent lack of adequate sleep increases the risk of mental fatigue, physical exhaustion, fluctuations in BMI, and physical fitness decline [2-5]. A study by Chang and Chen (2015) examined the relationship between poor sleep quality, BMI, and aerobic fitness in college freshmen. The study involved 2011 college freshmen and used the Pittsburgh Sleep Quality Index (PSQI) to assess sleep quality. Participants were categorized into underweight, normal weight, and overweight groups using BMI ranges. Fitness tests were used to measure health status. The results showed that most participants were affected by poor sleep quality, with females being notably poorer sleepers and experiencing shorter physical tests. No significant difference was found between males and females regarding BMI scores. A correlation was found between poor sleep quality and higher BMI. In conclusion, poor sleep quality and decreased total sleep time contributed to higher BMI and lower physical fitness performance among college freshmen [5].

Sleep hours might be a significant regulator of BMI and metabolism. A study by Taheri et al. (2004) found a U-shaped curvilinear association between sleep time and BMI. In individuals sleeping less than eight hours, decreased sleep was proportional to increased BMI. Deprived sleep was associated with lower leptin and higher ghrelin, independent of BMI. These changes in appetite-regulating hormones may explain the weight gain observed with sleep deprivation. The study suggests that these hormones may act upon increasing appetite, potentially explaining the weight gain observed with sleep deprivation [6].

A study by Grandner et al. (2015) found that fewer sleep hours can lead to obesity and cardiometabolic disease. The study used the National Health and Nutrition Examination Survey (NHANES) and 5,607 participants aged over 16 years. The results showed a significant interaction between continuous sleep duration and categorical sleep hours. Younger participants had a pseudo-linear relationship, with the highest BMI associated with the shortest sleep duration. Middle-aged participants had a U-shaped relationship, while the oldest participants had a weaker relationship [7].

In contrast to Western cultures, Saudi Arabia, specifically, and Arab countries in general have a culture that does not encourage adequate nightly sleep duration. A study by Merdad et al. (2014) that enrolled 1035 adolescent students, ages 14-23 years, revealed that students slept an average of seven hours on school nights, with a 2.8- and six-hour delay in weekend sleep and rising times. Around one in every ten students stayed up all night and slept after school (depicting a reversed sleep cycle), which was more prevalent among boys and those with lower grade point averages. School type, stress, napping, and caffeine were predictors of excessive daytime sleepiness. Moreover, Bahammam (2011) highlighted the issue that the majority of the general population is uninformed of the health risks and adverse impacts linked with inadequate sleep and disruptions in biological rhythms [8,9].

Our study aims to evaluate sleep quality utilizing the PSQI and examine the association between poor sleep quality and high BMI, body weight fluctuations, and cognitive effects (such as difficulty concentrating) over a one-month period among university students attending Imam Mohammad Ibn Saud Islamic University (IMSIU) in Riyadh, Saudi Arabia. The importance of our study is to offer valuable insights into the role of weight conditions and cognitive difficulties as significant contributors to sleep disturbances and promote healthier lifestyles among university students.

## Materials and methods

Study design

This cross-sectional study was conducted on students attending Imam Mohammad Ibn Saud Islamic University (IMSIU) in Riyadh, Saudi Arabia, from September 2021 to May 2023 (99.3% of the response data were gathered during 2021; research activities for this study were temporarily halted due to COVID-19 restrictions). All participants provided informed consent before the questionnaire was administered, and the study's goal was explicitly explained. The data were gathered via an online questionnaire.

Study population

The study population included students aged 17 and above. All students attending IMSIU in Riyadh across all disciplines and academic levels were eligible to participate. Students from other universities were excluded. The participants were selected using convenience sampling, resulting in a sample size of 1297. The sample size was computed using a sample size calculation with a 95% confidence level and a 5% margin of error, as per the Raosoft sample size calculator (Raosoft, Inc., Seattle, WA).

Data collection and measures

Each participant was asked to fill out a self-administered online questionnaire. The survey comprised 27 questions, a combination of Likert-type and open-ended questions. The survey included basic demographic characteristics (i.e., age, gender, height, weight, college, and academic level) and a validated Arabic version of the PSQI, which is a self-report questionnaire that assesses sleep quality over a one-month time interval (Appendix 1). The PSQI consists of 19 questions grouped into seven component scores that measure subjective sleep quality, sleep latency, sleep duration, habitual sleep efficiency, sleep disturbance, use of sleep medicines, and daytime dysfunction (Appendix 2). These component scores can be added together to provide a global sleep quality score, with larger scores indicating greater sleep impairment; a cutoff score of five distinguishes between people with "good" and "poor" sleep (Buysse et al., 1989) [10]. Moreover, four additional subjective questions were provided, including following any weight loss regimen during the last month, experiencing weight loss or weight gain during the last month, and experiencing cognitive effects such as memory problems and difficulty concentrating during the last month (Appendix 3). The survey was distributed in September 2021 to all university students across all disciplines at IMSIU via the university email service.

Statistical analysis

Categorical variables were shown as counts and proportions (%), while continuous variables were displayed as means and standard deviations. The relationship between poor sleep quality among the students' basic demographic characteristics and experiencing cognitive difficulties and weight changes during the last month has been conducted using the chi-square test. Based on the significant results, a multivariate analysis was subsequently performed to determine the significant independent predictors of poor sleep quality with a corresponding odds ratio as well as a 95% confidence interval. Statistical significance was measured using p<0.05. All statistical data were analyzed using IBM SPSS Statistics for Windows, Version 26 (Released 2019; IBM Corp., Armonk, New York, United States).

Ethical considerations

The study was approved by the Institutional Review Board (IRB) of Imam Mohammad Ibn Saud Islamic University (Approval Number: 118/2020). Informed consent was obtained electronically before participants completed the survey. Participation was voluntary, and responses were anonymized to ensure confidentiality.

## Results

This study enrolled 1297 IMSIU students from various disciplines under science and humanities. As seen in Table 1, 48% were aged between 17 and 20 years old, with female students being dominant (70.8%). Regarding BMI, 13.6% were seen to be obese.

**Table 1:** Basic demographic characteristics of the Imam Mohammad Ibn Saud Islamic University (IMSIU) students (n=1297)

Study variables	N (%)
Age group	
17 - 20 years	623 (48.0%)
21 - 25 years	560 (43.2%)
>25 years	114 (08.8%)
Gender	
Male	379 (29.2%)
Female	918 (70.8%)
BMI level	
Underweight (<18.5 kg/m^2^)	208 (16.0%)
Normal (18.5 – 24.9 kg/m^2^)	673 (51.9%)
Overweight (25 – 29.9 kg/m^2^)	240 (18.5%)
Obese (≥30 kg/m^2^)	176 (13.6%)
College	
Preparatory Programs	217 (16.7%)
College of Economics and Administrative Sciences	320 (24.7%)
College of Media and Communication	118 (09.1%)
College of Computer and Information Sciences	123 (09.5%)
College of Languages and Translation	147 (11.3%)
College of Medicine	34 (02.6%)
College of Sharia	125 (09.6%)
College of Social Sciences	89 (06.9%)
College of Science	44 (03.4%)
College of Engineering	17 (01.3%)
College of Law	13 (01.0%)
Others	50 (03.9%)

Table 2 shows the detailed and global PSQI scores. It can be observed that the prevalence of poor sleep was 85.3%, and the rest were good sleepers (14.7%). The global PSQI mean score was 9.21 (SD 3.33). Regarding PSQI domains, sleep latency showed the highest mean score (mean: 1.92), followed by subjective sleep quality (mean: 1.68) and use of sleep medication (mean: 1.33), while daytime dysfunction showed the lowest mean score (mean: 0.88).

**Table 2:** Prevalence of poor sleep using the Pittsburgh Sleep Quality Index (PSQI) (n=1297)

PSQI domain	Mean ± SD
Sleep latency	1.92 ± 1.00
Subjective sleep quality	1.68 ± 0.91
Sleep disturbance	1.33 ± 0.58
Use of sleep medication	1.30 ± 0.91
Sleep efficiency	1.09 ± 0.78
Sleep duration	1.02 ± 1.07
Daytime dysfunction	0.88 ± 0.82
Global PSQI score	9.21 ± 3.33
Sleep Quality	
Poor sleep (score >5)	1106 (85.3%)
Good sleep (score ≤5)	191 (14.7%)

In Table 3, 19.4% were following a weight loss regimen over the last month. Only 4.9% strongly agreed about weight loss after a persistent lack of sleep, 6.6% strongly agreed that they gained weight due to persistent lack of sleep, and 26.4% strongly agreed that it affects their cognitive abilities.

**Table 3:** Cognitive and weight conditions (n=1297)

Statement	N (%)
Over the past month, have you followed any weight loss regimen?	
No	1046 (80.6%)
Yes	251 (19.4%)
Over the past month, have you experienced weight loss after a persistent lack of sleep?	
Strongly disagree	316 (24.4%)
Disagree	414 (31.9%)
Neutral	363 (28.0%)
Agree	141 (10.9%)
Strongly agree	63 (04.9%)
Over the past month, have you gained weight after a persistent lack of sleep?	
Strongly disagree	372 (28.7%)
Disagree	377 (29.1%)
Neutral	292 (22.5%)
Agree	170 (13.1%)
Strongly agree	86 (06.6%)
During the past month, did you experience cognitive effects (such as memory problems and difficulty concentrating)?	
Strongly disagree	105 (08.1%)
Disagree	140 (10.8%)
Neutral	265 (20.4%)
Agree	444 (34.2%)
Strongly agree	343 (26.4%)

In Table 4, sleep disturbance was significantly more common among students who were overweight or obese (p=0.017), those who strongly agreed/agreed of experiencing weight loss over the past month (p<0.001), those who strongly agreed/agreed of experiencing weight gain over the past month (p<0.001), and those who strongly agreed/agreed of experiencing cognitive effects over the past month (p<0.001).

**Table 4:** Factors that influence sleep quality among Imam Mohammad Ibn Saud Islamic University (IMSIU) students (n=1297) † University students who answered "Neutral" were excluded from the statistical test; § P-value has been calculated using the chi-square test; ** significant at p<0.05 level.

Factor	Poor sleep quality		P-value ^§^
	Yes N (%) ^(n=1106)^	No N (%) ^(n=191)^	
Age group			
17 - 20 years	530 (47.9%)	93 (48.7%)	0.844
>21 years	576 (52.1%)	98 (51.3%)	
Gender			
Male	322 (29.1%)	57 (29.8%)	0.838
Female	784 (70.9%)	134 (70.2%)	
BMI level			
Normal or underweight (<25 kg/m^2^)	737 (66.6%)	144 (75.4%)	0.017 **
Overweight or obese (≥25 kg/m^2^)	369 (33.4%)	47 (24.6%)	
Over the past month, have you followed any weight loss regimen?			
No	898 (81.2%)	148 (77.5%)	0.231
Yes	208 (18.8%)	43 (22.5%)	
Over the past month, have you experienced weight loss after a persistent lack of sleep? ^†^			
Strongly disagree/Disagree	590 (75.6%)	140 (90.9%)	<0.001 **
Strongly agree/Agree	190 (24.4%)	14 (09.1%)	
Over the past month, have you gained weight after a persistent lack of sleep? ^†^			
Strongly disagree/Disagree	607 (71.7%)	142 (89.3%)	<0.001 **
Strongly agree/Agree	239 (28.3%)	17 (10.7%)	
During the past month, did you experience cognitive effects (such as memory problems and difficulty concentrating)? ^†^			
Strongly disagree/Disagree	173 (19.3%)	72 (52.9%)	<0.001 **
Strongly agree/Agree	723 (80.7%)	64 (47.1%)	

In a multivariate regression model (Table 5), it was found that compared to university students who strongly disagreed/disagreed with experiencing weight loss, weight gain, or cognitive effects, university students who strongly agreed/agreed to experiencing weight loss over the past month were at a 4.8 times increased risk of sleep disturbance (AOR=4.829; 95% CI=2.017 - 11.558; p<0.001), students who experienced weight gain were at a 2.68-fold higher increased risk for a sleep disturbance (AOR=2.675; 95% CI=1.363 - 5.253; p=0.004), and students who experienced cognitive effects were at a 3.79 times increased risk for a sleep disturbance (AOR=3.788; 95% CI=2.383 - 6.023; p<0.001).

**Table 5:** Multivariate regression analysis to determine the significant independent risk factors of poor sleep quality among Imam Mohammad Ibn Saud Islamic University (IMSIU) students (n=1297) † University students who answered "Neutral" were excluded from the statistical test; AOR: adjusted odds ratio; CI: confidence interval; ** significant at p<0.05 level.

Factor	AOR	95% CI	P-value
BMI level			
Normal or underweight (<25 kg/m^2^)	Ref		
Overweight or obese (≥25 kg/m^2^)	1.212	0.727 – 2.020	0.460
Over the past month, have you experienced weight loss after a persistent lack of sleep? ^†^			
Strongly disagree/Disagree	Ref		
Strongly agree/Agree	4.829	2.017 – 11.558	<0.001 **
Over the past month, have you gained weight after a persistent lack of sleep? ^†^			
Strongly disagree/Disagree	Ref		
Strongly agree/Agree	2.675	1.363 – 5.253	0.004 **
During the past month, did you experience cognitive effects (such as memory problems and difficulty concentrating)? ^†^			
Strongly disagree/Disagree	Ref		
Strongly agree/Agree	3.788	2.383 – 6.023	<0.001 **

## Discussion

This study investigated the relationship between poor sleep quality and BMI that could negatively affect university students' performance at school. This study's findings revealed a significant relationship between poor sleep quality and BMI, indicating that students who were overweight/obese were more likely to exhibit sleep disorders than students who were in normal or underweight classes. This is consistent with the report of Bamania et al. [11]. Based on their reports, sleep duration was significantly associated with BMI. Obese students were found to have extremely poor sleep quality, according to PSQI scores. In a study done in the USA [3], adjusted for age and BMI, they found that women gained 1.14 kg intervals for sleeping five hours or less, more than those sleeping seven hours over 16 years. The relative risks for those sleeping five and six hours were 1.32 and 1.12 for a 15-kg weight gain, respectively. Interestingly, a study done at Qassim University [12] indicated that although they found a significant difference between sleep duration in terms of BMI, the overall sleep quality in relation to BMI did not yield statistical significance (p>0.05). Our findings provided evidence of the relationship between sleep quality and BMI subjected to clinical consideration and could help guide future research regarding the sleep disorder-obesity relationship.

According to the PSQI questionnaire, the prevalence of poor sleep quality (global PSQI score >5) among IMSIU students was 85.3%, and the rest had no sleep problems (14.7%). The overall mean score of PSQI was 9.21 (SD 3.33). Consistent with our results, a study conducted in Indonesia [13] documented that poor sleep quality was found in 82% of the students. However, in China [14], the sleep quality index was lower than in our reports. Indicating poor sleep quality was detected in 36.5% of the male and 39.1% of the female students (mean score: 4.91; SD 2.67). This has been concurred by the study done in India [15], with 38.1% having poor sleep quality (PSQI score >5) and 43.3% being sleep deprived with five to six hours of sleep duration.

Regarding PSQI domains, the most important domains contributing to poor sleep are sleep latency, followed by subjective sleep quality, sleep disturbance, use of sleep medication, sleep efficiency, and sleep duration, while daytime dysfunction has the least effects, as demonstrated in Table 2. According to the reports of Mirdha et al. [16], the most common contributing factors to poor sleep quality were restricted sleep duration, poor sleep efficiency, daytime dysfunction, long sleep latency, and sleep disturbances. They further emphasized that poor sleep quality is concomitant with many health conditions, and it can also lead to obesity, leading to an increased risk of diabetes mellitus (DM), cardiovascular disease (CVD), and some types of cancer.

Moreover, we identified that 18.5% of the students were overweight, and 13.6% were obese. We discovered that 19.4% had followed a weight loss regimen over the past month. Our results also disclosed that due to persistent lack of sleep, 15.8% strongly agreed/agreed that they experienced weight loss, while 19.7% experienced weight gain in the same scenario. As a result, 60.6% strongly agreed/agreed that this affected their cognitive abilities. Among the medical students surveyed by Alodhayani et al. [17], sleep disturbance has impacted weight gain, and habits such as lying down after lunch, sitting, or reading were at increased risk of developing obesity in the future.

Our study found no significant difference between students' ages in relation to sleep disorders. However, we noted that weight loss, weight gain, and cognitive condition due to a persistent lack of sleep were identified as significant independent risk factors for poor sleep quality. According to the study of Taheri et al. [6], an increase in BMI was proportional to a decrease in sleep. Respondents with less sleep had decreased leptin and increased ghrelin. These variations in leptin and ghrelin are likely to increase appetite, possibly describing the increased BMI seen with short sleep duration. However, in a study by Alhusseini et al. [18], moderate to severe anxiety was associated with underweight BMI, while higher PSQI scores were associated with obese and overweight respondents, suggesting that being overweight and obese had direct relations with anxiety and poor sleep quality. Incidentally, in the USA [19], controlling for age and sex, only sleep disturbances rather than sleep duration were associated with being overweight.

Limitations

Differences in gender ratio was a limitation in this study. The number of female participants who completed the survey exceeded the number of male participants. This is associated with the method of distribution, which makes it difficult to control the demographics of university students through an online questionnaire.

The timing of the questionnaire distribution was parallel with the COVID-19 crisis lockdown. Several factors may be contributing to increased sleep problems among students. These include increased stress and anxiety, changes in sleep routines, and increased screen time.

A key limitation of this study lies in its reliance on self-reported, subjective measures to evaluate experiences of weight loss, weight gain, and cognitive effects within the past month. These measures may be susceptible to recall bias and subjective interpretation. Future research could benefit from objective measures to improve the validity of these findings.

Confounding factors for poor sleep quality among students, such as stress, anxiety, and caffeine consumption, may hinder the sleep patterns among students, necessitating future research to include confounding factors.

## Conclusions

The incidence of poor sleep among university students was high. Increased BMI, weight loss, weight gain, and cognitive dysfunction due to lack of sleep were identified as the influential factors in poor sleep quality. This study highlights the need for targeted interventions to improve sleep hygiene and promote physical well-being programs in this population. The study reinforces the link between poor sleep quality and higher BMI among university students, highlighting weight fluctuations and cognitive difficulties as significant contributors to sleep disturbances.

## Figures and Tables

**Figure 1:**
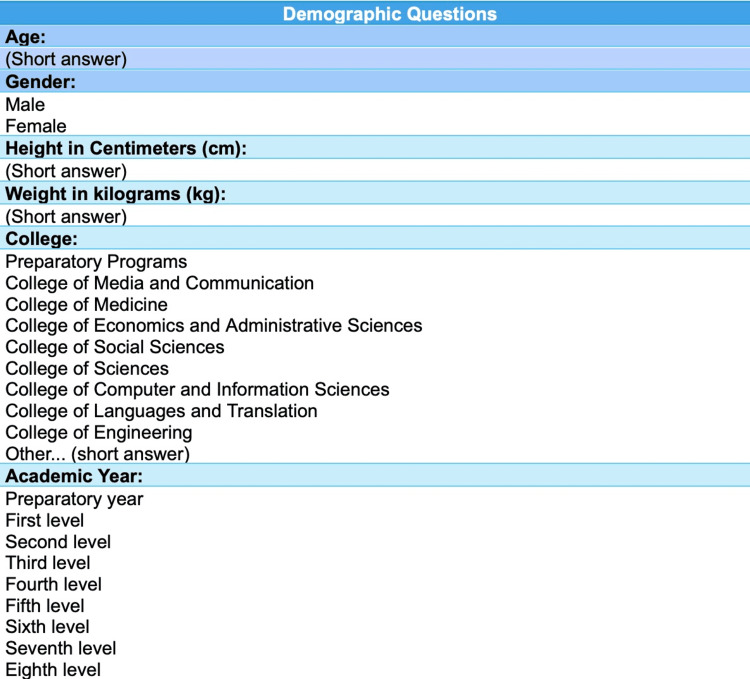
Demographic information

**Figure 2:**
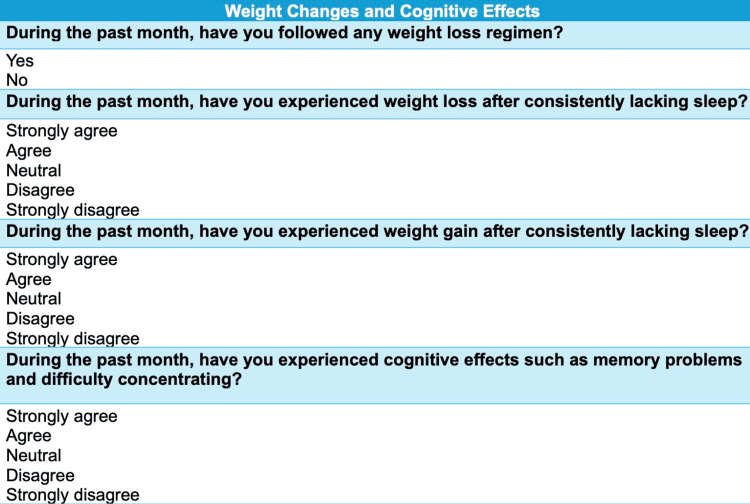
Weight changes and cognitive effects

## Appendices

Appendix 1

Appendix 2

**Table 6:** Pittsburgh Sleep Quality Index (PSQI)

Pittsburgh Sleep Quality Index (PSQI)				
1. During the past month, what time have you usually gone to bed at night?				
(Short answer)				
2. During the past month, how long (in minutes) has it usually taken you to fall asleep each night?				
(Short answer)				
3. During the past month, what time have you usually gotten up in the morning?				
(Short answer)				
4. During the past month, how many hours of actual sleep did you get at night? (This may differ from the number of hours you spent in bed.)				
(Short answer)				
5. During the past month, how often have you had trouble sleeping because you…	Not during the past month	Less than once a week	Once or twice a week	Three or more times a week
a. Cannot get to sleep within 30 minutes				
b. Wake up in the middle of the night or early morning				
c. Have to get up to use the bathroom				
d. Cannot breathe comfortably				
e. Cough or snore loudly				
f. Feel too cold				
g. Feel too hot				
h. Have bad dreams				
i. Have pain				
j. Other reason(s), please describe:				
6. During the past month, how often have you taken medicine to help you sleep (prescribed or over the counter)?				
7. During the past month, how often have you had trouble staying awake while driving, eating meals, or engaging in social activity?				
	No problem at all	Only a very slight problem	Somewhat of a problem	A very big problem
8. During the past month, how much of a problem has it been for you to keep up enough enthusiasm to get things done?				
	Very good	Fairly good	Fairly bad	Very bad
9. During the past month, how would you rate your sleep quality overall?				
	No bed partner or roommate	Partner/roommate in another room	Partner in same room but not same bed	Partner in same bed
10. Do you have a bed partner or roommate?				
	Not during the past month	Less than once a week	Once or twice a week	Three or more times a week
If you have a roommate or bed partner, ask him/her how often in the past month you have had:				
a. Loud snoring				
b. Long pauses between breaths while asleep				
c. Legs twitching or jerking while you sleep				
d. Episodes of disorientation or confusion during sleep				
e. Other restlessness while you sleep, please describe:				

Appendix 3
